# Effect of psoralen on the regulation of osteogenic differentiation induced by periodontal stem cell-derived exosomes

**DOI:** 10.1007/s13577-023-00918-2

**Published:** 2023-06-03

**Authors:** Jie Yu, Xiaonan Wu, Wenyi Zhang, Fuhang Chu, Qi Zhang, Meihua Gao, Yingjie Xu, Yingtao Wu

**Affiliations:** 1 School of Stomatology, Qingdao University, Qingdao, 266023 China; 2 Qingdao Stomatological Hospital Affiliated to Qingdao University, No.17 Dexian Road, Shinan District, Qingdao, 266001 Shandong China

**Keywords:** Human periodontal stem cells, Psoralen, Exosomes, hsa-miR-125b-5p, Osteogenesis

## Abstract

Periodontitis is a chronic inflammatory disease that is the main cause of tooth loss in adults, and the key to periodontitis treatment is the repair and regenerate of periodontal bone tissue. Psoralen is the main component of the *Psoralea corylifolia* Linn, which shows antibacterial, anti-inflammatoryand osteogenic activities. It promotes the differentiation of periodontal ligament stem cells toward osteogenesis. Exosomes secreted by stem cells play important roles in information transmission during the osteogenic differentiation process. The aim of this paper was to investigate the role of psoralen in regulating osteogenic miRNA information in periodontal stem cells and in periodontal stem cells exosomes and the specific mechanism of its action. Experimental results show that exosomes of human periodontal ligament stem cell origin treated with psoralen (hPDLSCs + Pso-Exos) were not significantly different from untreated exosomes (hPDLSC-Exos) in terms of size and morphology. Thirty-five differentially expressed miRNAs were found to be upregulated and 58 differentially expressed miRNAs were found to be downregulated in the hPDLSCs + Pso-Exos compared to the hPDLSC-Exos (*P* < 0.05). hsa-miR-125b-5p was associated with osteogenic differentiation. Among them, hsa-miR-125b-5p was associated with osteogenic differentiation. After hsa-miR-125b-5p was inhibited, the osteogenesis level of hPDLSCs was enhanced. In summary, the osteogenic differentiation of hPDLSCs was promoted by psoralen through the downregulation of hsa-miR-125b-5p gene expression in hPDLSCs, and the expression of the hsa-miR-125b-5p gene was also downregulated in exosomes. This finding provides a new therapeutic idea for using psoralen to promote periodontal tissue regeneration.

**Supplementary Information:**

The online version contains supplementary material available at 10.1007/s13577-023-00918-2.

## Introduction

Periodontitis is an inflammatory disease caused by bacteria that leads to the loss of periodontal hard tissue and is the leading cause of tooth loss in adults [1, 2]. The key to the treatment of periodontitis is the repair and regeneration of periodontal bone tissue [3]. Strategies to repair bone defects caused by periodontitis are key topics in the field of oral cavity research. Periodontal ligament stem cells (PDLSCs) are isolated from human periodontal ligament tissue. They show the potential for self-renewal and multidirectional differentiation [3]. Therefore, they are important cells involved in the repair of periodontal tissue damage. As the main sources of newly attached cells after periodontitis treatment, they can differentiate into osteoblasts [4]. Promoting osteogenic differentiation of PDLSCs is currently a research hotspot in the field of periodontal bone tissue repair and regeneration.

Psoralen is a weak estrogen-like compound extracted from the ripe fruit of the leguminous plant *Psoralea corylifolia* Linn, and it has shown anti-osteoporotic immune, antitumor, antibacterial, anti-inflammatory, and antioxidant activities [5–7]. It leads to the return to the kidney and spleen meridian, warming the kidney and supporting yang, relieving qi and asthma, warming the spleen and stopping diarrhea. It is an important component of classic prescriptions such as the Ershen pill and Qing'e pill and is used for the treatment of osteoporosis, vitiligo and other diseases [8]. Preliminary animal experiments have shown that psoralen can reduce alveolar bone resorption in rats with periodontitis [9]. Psoralen can also promote osteogenic differentiation of PDLSCs [10], but its specific mechanism of action and information on its role in osteogenesis remain to be further explored.

Exosomes play roles in information transmission by carrying and transferring important biologically active molecules such as proteins, lipids, and nucleic acids between cells and are important to the paracrine mechanism [11]. Because exosomes carry important biological information, such as the proteins and nucleic acids derived from the source cells, they can exert all or some of the functions of the source cells. For example, stem cell-derived exosomes can mediate bone repair and regeneration [12]. Research suggests that the mechanism by which exosomes promote bone regeneration is related mainly to the regulatory role played by microRNAs (miRNAs) carried in each stage of osteogenesis [13].

Exosomes derived from bone marrow mesenchymal stem cells (MSCs) not only can affect MAPK, Wnt/β-catenin and other osteogenesis-related signaling pathways but also regulate the osteogenic differentiation of bone marrow-derived mesenchymal stem cells (BMSCs) through their miRNAs, long-encoding RNAs (lncRNAs) and other substances [14]. Studies have shown that miRNAs, as important regulatory factors, play key role in bone metabolism processes such as the proliferation, differentiation, metabolism and apoptosis of osteoblast-related cells [15–17]. Among these miRNAs, miR-125b is related to osteogenesis and can negatively regulate the osteogenic differentiation of human (h) BMSCs [18]. Studies have shown that hsa-miR-125b-5p, a member of the miR-125b family, is upregulated in a high-fat environment, resulting in an inhibitory effect on osteogenesis, but the regulation of hsa-miR-125b-5p in PDLSCs and the mechanism of osteogenic differentiation still need to be explored.

High-throughput sequencing revealed that the level of hsa-miR-125b-5p was reduced in exosomes derived from periodontal ligament stem cells after psoralen treatment. Therefore, we speculate that psoralen promotes the osteogenic differentiation of PDLSCs by downregulating the expression of hsa-miR-125b-5p and can transmit osteogenesis-related information through exosomes to promote the repair and regeneration of periodontal bone tissue. This study provides new ideas for the treatment and prognosis of periodontitis.

## Materials and methods

### Cell culture

Human periodontal ligament stem cells were isolated from the periodontal ligament of healthy third molars (without any dental and periodontal tissue disease). The third molars used were all from patients (18–25 years old) without systemic diseases.

All the patients came to Qingdao Stomatological Hospital for oral and maxillofacial surgery and signed the informed consent. This study was examined by the Ethics Committee of Qingdao Stomatological Hospital affiliated to Qingdao University (2021KQYX030).

HPDLSCs were extracted from the tissue block, and the 3–6 generation hPDLSCs were used in this study. According to different experiments, different concentrations of fetal bovine serum (FBS, Procell, Wuhan, China) and 1% penicillin–streptomycin were added into α-MEM medium to culture hPDLSCs. (Biological Industries, Israel). All cells were cultured at 37 ℃, 5% CO_2_ constant humidity condition.

### Adipogenic differentiation

HPDLSCs (2 × 10^5^ cells/well) were seeded into six-well plates, cultured with adipogenic differentiation induction medium (Procell, Wuhan, China), and the medium was changed every 3 days for 21 days. 4% paraformaldehyde (Elabscience, Wuhan, China) was used to fix cells, stained using Oil Red O after washing with PBS (Procell, Wuhan, China), then observed under an optical microscope (OLYMPUS, Japan).

### Osteogenic differentiation

HPDLSCs (2 × 10^5^ cells/well) were seeded into six-well plates, cultured with osteogenic differentiation induction medium (Procell, Wuhan, China), and the medium was changed every 3 days for 21 days, fixed with 4% paraformaldehyde, stained using alizarin red after washing with PBS, then observed under an optical microscope.

### Flow identification

HPDLSCs positive markers (CD105, CD73, and CD90) and negative markers (CD34, and CD45) were identified using flow cytometry. HPDLSCs (P4) were digested and centrifuged, then the cell density was adjusted to 1 × 10^4^ cells/mL. 100 μL cell suspension was added into centrifuge tubes, then mouse anti-human antibodies CD34 (E-AB-F1143C), CD45 (E-AB-F1137C), CD105 (E-AB-F1143D), CD73 (E-AB-F1242D), and CD90 (E-AB-F1167D) (Elabscience, Wuhan, China) (1:50) were added, respectively, PBS was added as the control group, and all cells were incubated at room temperature for 20 min in the dark. The supernatant was discarded by centrifugation, and 500 μL PBS was added to resuspend the cells after washing twice. Then flow cytometry was used to detect the expression levels of different antibodies (DxFLEX, Beckman Coulter, Suzhou, China).

### Cell viability detection

HPDLSCs were inoculated in 96-well plates (5 × 10^3^ cells/well) and cultured in complete medium for 24 h. After the original medium was discarded, the cells were cultured with complete medium containing different concentrations of psoralen (0 μg/mL, 5 μg/mL, 10 μg/mL, 15 μg/mL, 20 μg/mL, dissolved in DMSO)(HPLC ≥ 98%, wavelength 280 nm) (Solarbio, Beijing, China). Each concentration was set up with 5 replicate wells. After culturing for 1 day, 3 days, 5 days, and 7 days, the cell counting kit 8 (CCK-8, Absin Bioscience Inc., Shanghai, China) was used to detect the cell viability of each well. Then the absorbance was determined at a wavelength of 450 nm with a microplate reader (SynergyH1/H1M, Bio-Tek, China).

## Detection of the osteogenic ability of psoralen

### Alizarin red detection

HPDLSCs (2 × 10^5^ cells/well) were seeded into six-well plates and cultured using a complete medium containing 10 μg/mL psoralen. The blank control group was cultured with a medium without psoralen, and the positive control group was cultured with an osteogenic medium for 21 days. Fixed with 4% paraformaldehyde, stained using alizarin red after washing with PBS, then observed under an optical microscope. After the images were collected, cetylpyridinium chloride solution was added into the wells to dissolve the calcium nodules at room temperature, then the decolorization solution was collected to measure the absorbance at 562 nm with a microplate reader.

### ALP staining detection

HPDLSCs (2 × 10^5^ cells/well) were inoculated in six-well plates; the blank control group was cultured with simple medium, the positive control was cultured with an osteogenesis medium, the psoralen group was cultured using a complete medium containing 10 μg/mL psoralen for 21 days, fixed with 4% paraformaldehyde for 30 min, stained using ALP-modified Gomori calcium-cobalt method (Solarbio, Beijing, China), and observed under an optical microscope.

### Quantitative detection of ALP

HPDLSCs (2 × 10^5^ cells/well) were inoculated in six-well plates’ the blank control group was added with a simple medium, the positive control was cultured with an osteogenesis medium, and the psoralen group was cultured using a complete medium containing 10 μg/mL psoralen for 21 days. All cells were lysed using RIPA solution on ice (Elabscience, Wuhan, China). Then the lysate was centrifuged at 12,000 rpm, 4 ℃. The protein concentrations of different groups were measured using a BCA kit (Elabscience, Wuhan, China). Then, the absorbance was detected using a microplate reader at a wavelength of 520 nm. ALP activity was calculated according to the formula as follows:ALP activity (gold unit /gprot) = (△A-b)$\div$ a $\div$ C_pr_ × f (△A:Measuring hole OD value–blank OD value;f: Dilution ratio before the sample is added to the detection system; C_pr_: Protein concentration of sample to be tested).

### Exosome extraction and identification

#### Exosome extraction

The third-generation hPDLSCs were placed in 75 mm^2^ culture flask, and psoralen group was cultured with 10 μg/mL psoralen for 72 h and then amplified. When the fourth-generation cells grew to 80% of the culture bottle, the supernatant was taken from the exosome-free serum after 48 h starvation culture. The above operations were repeated on the blank control group, except that the culture medium did not contain psoralen. The cell supernatants were centrifugated at 4 ℃ in a gradient (300 g, 3000 g). Then discarded the pellet; the supernatants were centrifugated again at 4 ℃ in gradients 300 g, 2000 g, 10,000 g, and 100,000 g after rebalancing. The final transparent precipitate was taken and resuspended with PBS.

#### Identification of exosomes

The particles collected above were identified using nanoparticle tracking analysis (NTA), transmission electron microscopy (TEM), and western blot. 15 μL exosome samples were placed on the copper grid for 1 min, then stained using 2% uranyl acetate at room temperature for 1 min. The dyed samples were baked under the lamp for 10 min, observed, and photographed under a transmission electron microscope (Hitachi, HT-7700).

After cleaning the sample pool with ultrapure water, polystyrene microspheres (100 nm) were used to calibrate the instrument (Nano Sight NS300, Malvern, NS300), and the sample pool was washed with 1 × PBS buffer (SH30028.02, Hy Clone); exosome samples were tested after diluting in PBS buffer.

Exosome-specific proteins Calnexin (ab22595), Syntenin-1(ab19903), and TSG101(ab125011) (Abcam, Cambridge, Britain) were detected by western blot. The exosomes were lysed using RIPA and the concentrations were determined using a BCA kit. 12% SDS PAGE electrophoresis gel was used to separate protein samples; then the protein was transferred onto a PVDF membrane. The blotted membrane was blocked in 5% skimmed milk TBST for 1 h, then incubated with Calnexin and Syntenin-1 primary antibodies at 4 °C overnight (1:5000). After washing the membrane, incubated the corresponding secondary antibody (1:5000) for 1 h at room temperature, washed the membrane again, and added exposure solution to expose the bands.

### High-throughput MicroRNA sequencing

High-throughput sequencing services and subsequent bioinformatics analysis were provided by Heyuan Biotechnology (China, Shanghai). The small RNA sequencing library was prepared using the TruSeq Small RNA Sample Prep Kits (Illumina, San Diego, USA) kit. After the library was constructed, the library was sequenced using Illumina Hiseq2000/2500, and the sequencing read length was single-end 1 × 50 bp. The miRNA data were analyz****ed using ACGT101-miR, and the difference expression of miRNAs between samples was compared using the T-test, a P value less than 0.05 was considered statistically significant.

### Inhibitor production and transfection

The differential gene hsa-miR-125b-5p mature body sequence information TCCCGGAGACCCTAACTTGTGA was provided to the inhibitor production company (Ribobio, Guangzhou, China) to make the hsa-miR-125b-5p gene inhibitor.

HPDLSCs (2 × 10^5^ cells/well) were planted into six-well plates and incubated for 24 h; then the original medium was discarded and the control group was added with hsa-miR-125b-5p negative control gene (NC), and the experimental group was incubated with hsa-The miR-125b-5p gene inhibitor in the dark for 24 h. Then all groups were observed under a fluorescent microscope; green fluorescence indicates successful transfection.

### The effect of hsa-miR-125b-5p on the osteogenic ability of hPDLSCs

#### ALP qualitative detection

HPDLSCs (2 × 10^5^ cells/well) were seeded into six-well plates. The blank control group was added with simple medium, and the experimental group was added with 10 μg/mL psoralen, inhibitor + 10 μg/mL psoralen (psoralen + inhibitor), and inhibitor respectively for 7 days. Then the cells were fixed with 4% paraformaldehyde for 30 min, stained using ALP-modified Gomori calcium-cobalt method (Solarbio, Beijing, China), and photographed for observation.

#### Quantitative detection of ALP

HPDLSCs (2 × 10^5^ cells/well) were seeded in 6-well plates as mentioned above. After that, all cells were lysed with RIPA on ice and centrifuged at 4 °C, 12,000 rpm. The protein concentrations were determined using a BCA kit. Then, the absorbance was measured to calculate ALP activity.

### RT-PCR reaction

Total RNA was extracted from all groups mentioned above, and RNA concentration was assessed using a micro drop ultra-microspectrophotometer Micro Drop (Bio DL, Texas, USA). ALL samples used GAPDH (Abcam, Cambridge, Britain) as an internal reference, and the mRNA expression levels of RUNX2 (Abcam, Cambridge, Britain), OCN (Abcam, Cambridge, Britain), and ALP (Abcam, Cambridge, Britain) were quantified 2^−△△CT^ method. The primers used were RUNX2:150 AGG CAG TTC CCA AGC ATT TCA TCC (forward) and 150 TGG CAG GTA GGT GTG GTA GTG AG (reverse); OCN:135 AGG GCA GCG AGG TAG TGA AGA G (forward) and 135 GGT CAG CCA ACT CGT CAC AGT C (reverse); ALP:92 ACT CTC CGA GAT GGT GGT GGT G (forward) and 92 CGT GGT CAA TTC TGC CTC CTT CC (reverse).

### Western blot detection

The total cellular protein from the groups mentioned above was extracted using the RIPA lysis buffer, and the protein concentration was determined using a BCA method. Then the protein was separated by SDS-PAGE and electro-transferred to PVDF membranes and incubated with RUNX2 (ab114133) and ALP (ab229126) (Abcam, Cambridge, Britain) primary antibody (1:5000) overnight at 4℃. The HRP-labeled protein bands were exposed by an ECL kit after washing with TBST and then photographed. β-actin was used as the internal reference, and the relative protein expression was analyzed by Image J 2.0 software.

### Statistical Analysis

All data were expressed as mean ± SD. Two-tailed T-test was used for the comparison of the two groups. After the normal test, a one-way analysis of variance was used for the comparison of the arrays. Statistical analysis was performed using Graph Pad Prism 9 software (* *P* < 0.05, ** P < 0.01, and *** *P* < 0.001).

## Results

### Characterization of periodontal ligament stem cells (PDLSCs)

**Fig. 1:**
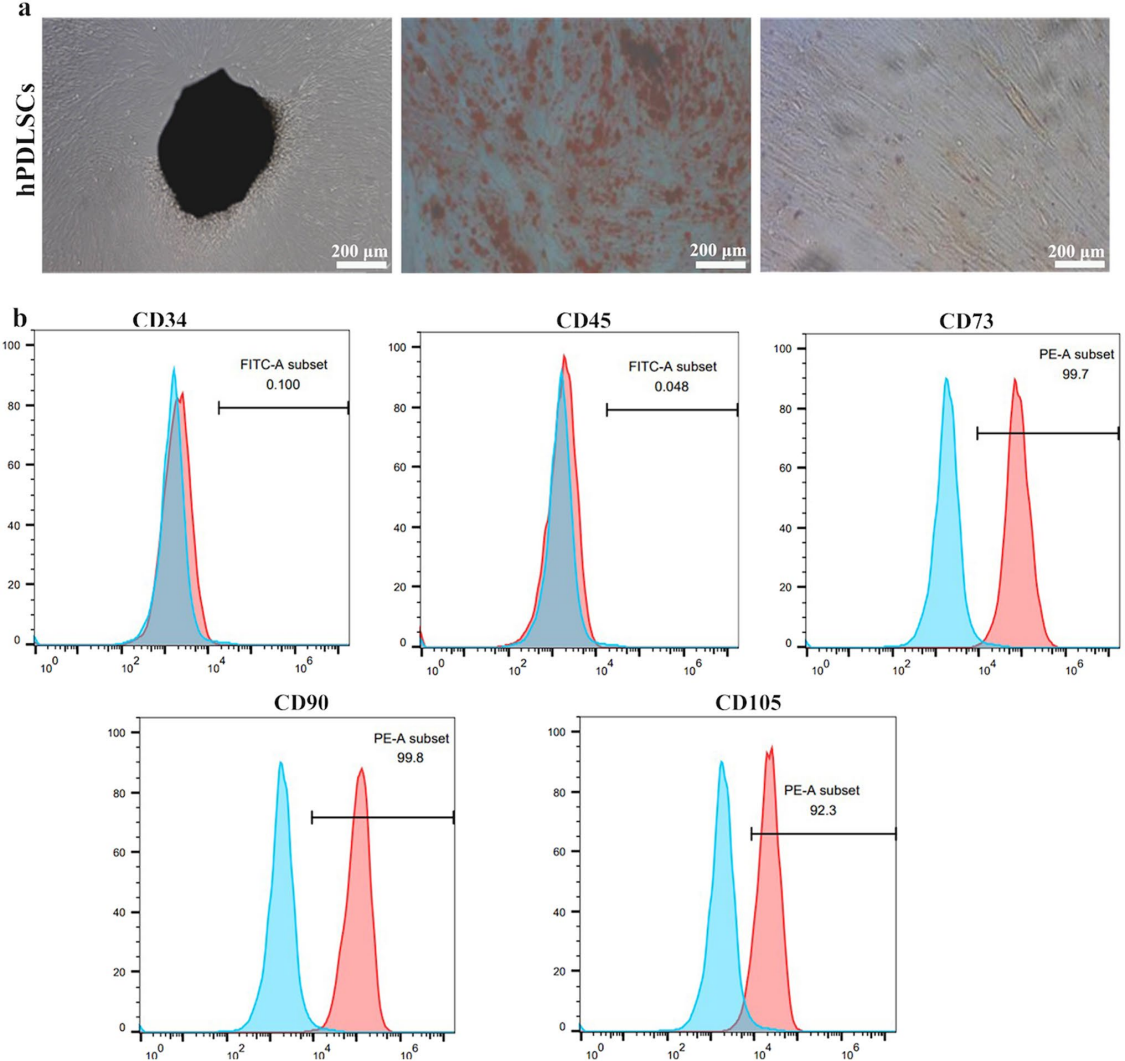
Identification of periodontal membrane stem cells **a**. Black represents a block of periodontal membrane tissue around which long spindle-shaped periodontal membrane stem cells can be seen; the red portion is a calcium nodule after alizarin red staining; the pink portion is a lipid droplet after oil red O staining. **b**. Identification of the antibodies on the surface of periodontal membrane stem cells

The PDLSCs exhibited a typical long spindle shape and were arranged in a vortex, as observed by microscopy. The results of osteogenic and adipogenic differentiation experiments showed that the cells exhibited osteogenic and adipogenic characteristics, and mineralized nodules and lipid droplets were observed (Fig. 1a). A flow cytometry analysis showed that the expression of CD73 was 99.7% on the surface of PDLSCs, CD90 expression was 99.8%, CD105 expression was 92.3%, which was significant, the antibody CD34 was expressed at 0.1% and CD45 was expressed at 0.048%, and the expression was negative (Fig. 1b). These results showed that the cells extracted from the experiment showed the basic characteristics of MSCs and were identified as periodontal stem cells, which were used in subsequent experiments.

### Effect of psoralen on cell viability

**Fig. 2:**
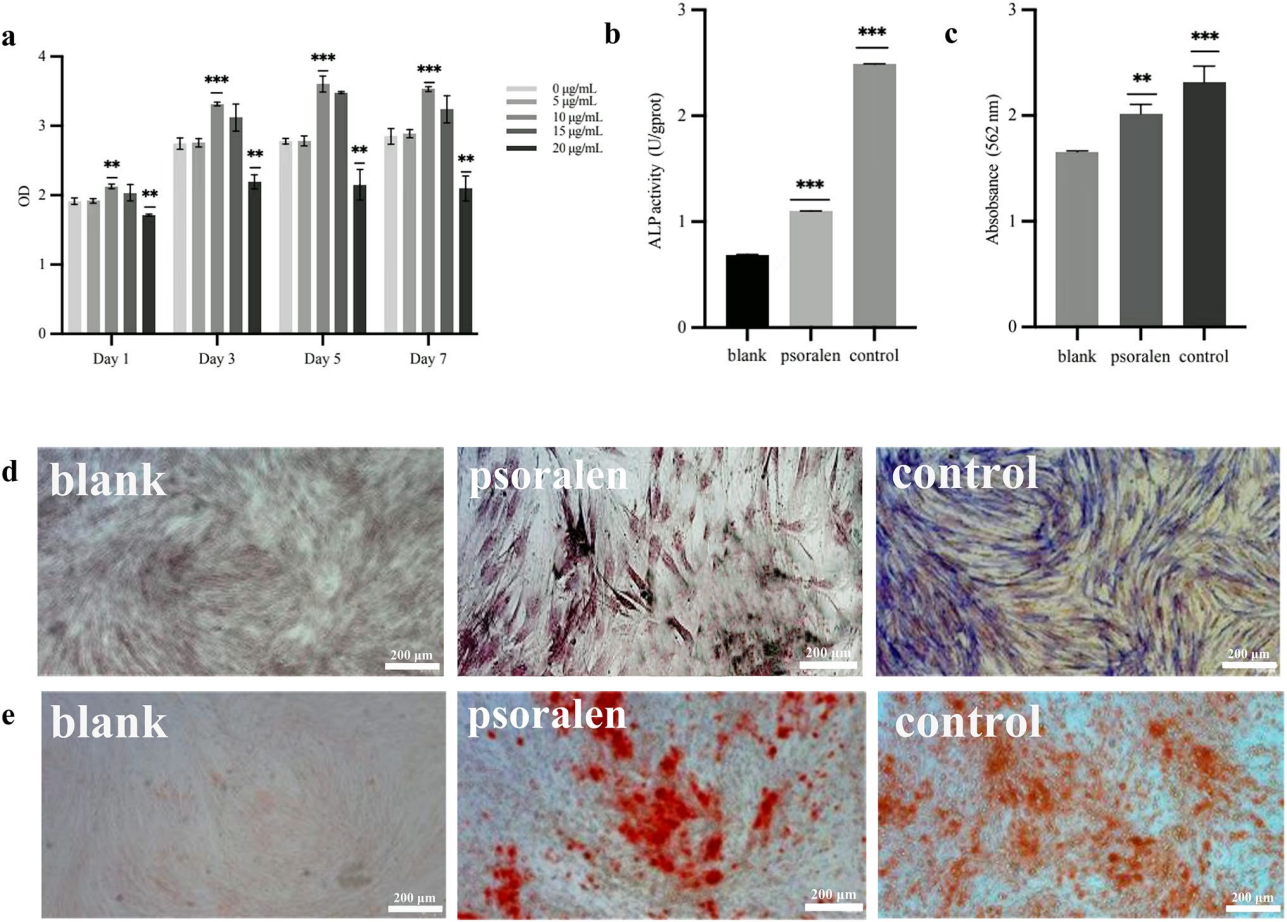
Osteogenic differentiation of hPDLSCs is promoted by psoralen **a**. Effect of psoralen on the activity of periodontal stem cells. **b**. Expression of ALP activity. (**P < 0.01, ***P < 0.001). **c**. Expression of alizarin red activity. (**P < 0.01, ***P < 0.001). **d**. ALP staining in the blank control group; blue‒black ALP staining in the psoralen group; blue‒black ALP staining in the osteogenic medium group Expression of ALP activity. **e**. Alizarin red staining in the blank control group; red calcium nodules are seen after alizarin red staining in the psoralen group; red calcium nodules are seen after alizarin red staining in the osteogenic medium group

The Cell-Counting Kit-8** (**CCK-8) assay was performed to detect different concentrations of psoralen (0, 5, 10, 15, and 20 μg/mL) used for treatments at different times (1, 3, and 5 days). The results of cell activity experiments after 7 days confirmed that, compared with the blank control group, 20 μg/mL psoralen exerted a significant inhibitory effect on PDLSCs within 7 days (*P* < 0.01), and 10 μg/mL psoralen promoted the proliferation of PDLSCs (*P* < 0.01). Other concentrations of psoralen exerted no statistically significant effect on PDLSCs. (Fig. 2a). Therefore, 10 μg/mL was determined to be the best concentration of psoralen for the experiments.

### Effect of psoralen on the osteogenesis of periodontal stem cells

Psoralen promotes the osteogenic differentiation of periodontal ligament stem cells. ALP staining revealed that both the psoralen group and the osteogenic induction medium group (Fig. 2b) were blue‒black, indicating that the psoralen and osteogenic induction medium groups showed obvious ALP production compared with the blank control group. The experimental results showed that the ALP activity in the psoralen group and the osteogenic induction medium group was higher than that in the blank control group (*P* < 0.001) (Fig. 2d). After 21 days in coculture, the three groups were stained with alizarin red, and red calcium nodules were seen in the psoralen treatment group and osteogenic induction medium group (Fig. 2c), indicating that psoralen and osteogenic induction medium had significantly more calcium nodules than the blank control group. Compared with those in the blank control group, the calcium nodules in the psoralen group (*P* < 0.01) and the osteogenic induction medium group (*P* < 0.001) were significantly higher than those in the blank control group (Fig. 2d). The experiments confirmed that psoralen administered at 10 μg/mL shows the ability to promote osteogenic differentiation of periodontal ligament stem cells.

### Identification of exosomes derived from human (h) PDLSCs

**Fig. 3:**
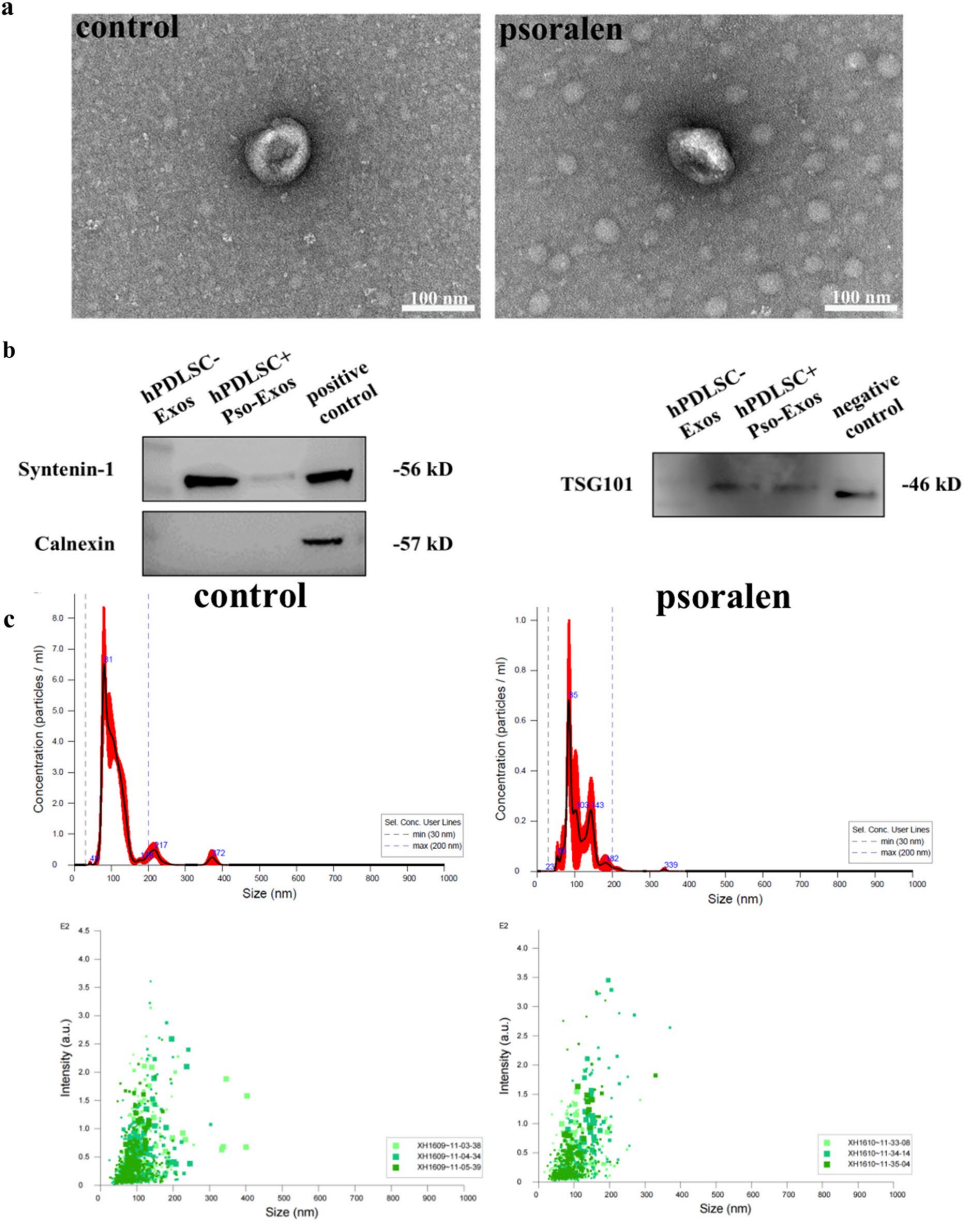
Dentification of exosomes **a**. Electron micrographs of exosomes from the control and psoralen groups. **b**. Western blot bands detected with anti-Syntenin-1 antibody, anti-Calnexin antibody and anti-TSG101. **c**. The particle size and density of exosome granule distribution in the control group and in the psoralen group

The transmission electron microscopy (TEM) results showed that both hPDLSC-exosomes (Exos) and hPDLSC-Exos treated with psoralen (hPDLSC + Pso-Exos) were round (Fig. 3a), which conformed to the morphological characteristics of exosomes. A Western blot analysis showed that the characteristic exosome surface marker Syntenin-1 of hPDLSC-Exos and hPDLSC + Pso-Exos was expressed, but Calnexin was not (Fig. 3b). Nanotracking analysis (NTA) measurements showed that these particles ranged in size from 30 to 200 nm, with an average particle size of 113.7 ± 9.4 nm in the control group and 112.2 ± 8.6 nm in the psoralen group. Particle size distribution and density are shown in Fig. 3c. In the control group, particle concentration in the distribution range of 30–200 nm is 2.89E + 09 particles/mL, accounting for 93.3%. The particle concentration of psoralen group was 2.25E + 09 particles/mL in the range of 30–200 nm, accounting for 98.3%.

### Differential gene expression in exosomes

**Fig. 4:**
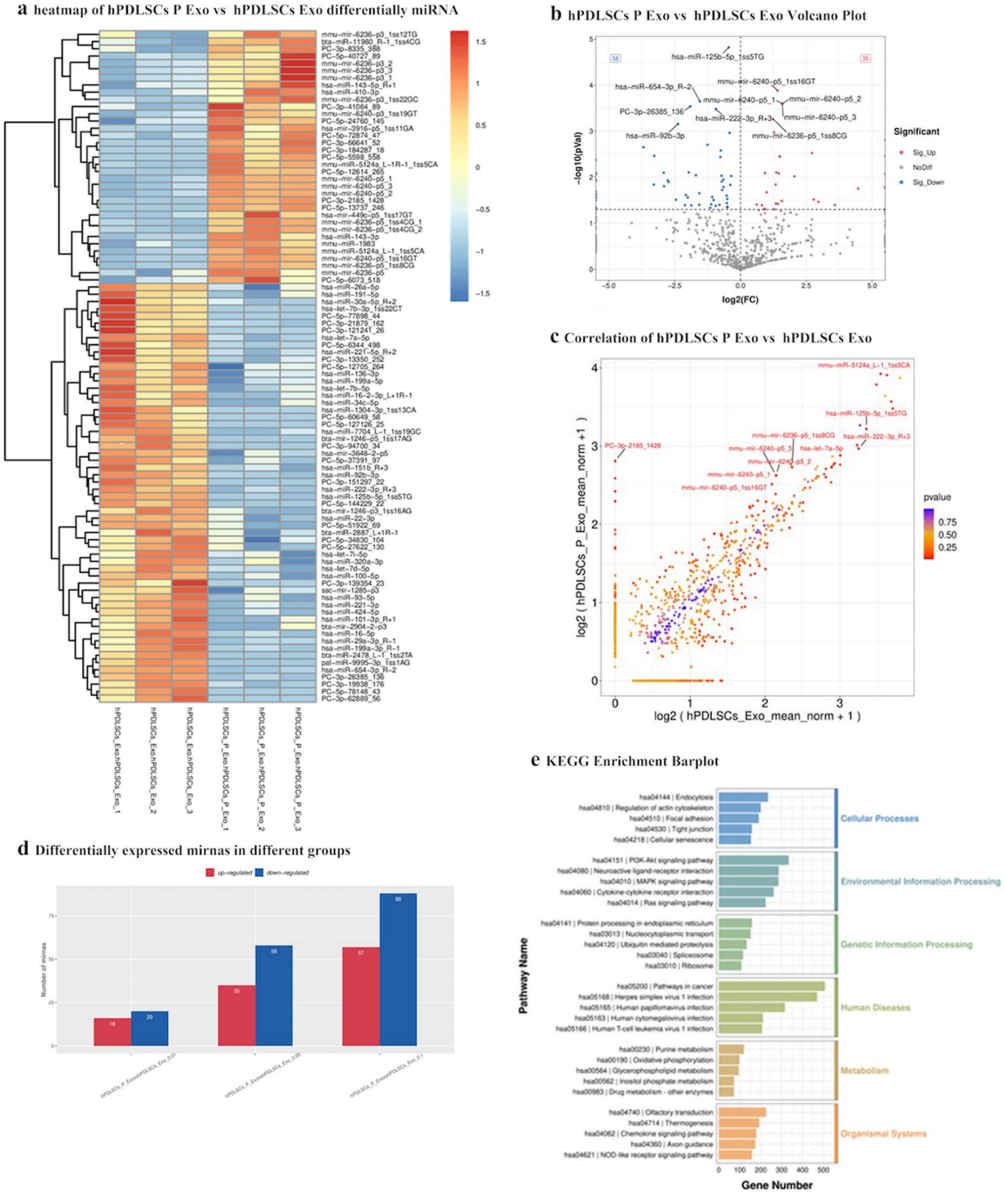
Differential gene expression analysis with hPDLSCs + Pso-Exos and hPDLSC-Exos **a** Heatmap showing differentially expressed miRNAs. **b**. **c**. Volcano map of differentially expressed miRNAs. **d**. Number of miRNAs that were up- or downregulated. **e**. KEGG functional enrichment analysis

Illumina HiSeq2000/2500 was used for sequencing to detect differential gene expression between hPDLSCs + Pso-Exos and hPDLSC-Exos. A total of 35 miRNAs were upregulated and 58 miRNAs were downregulated in hPDLSC + Pso-Exos compared to hPDLSC-Exos (*P* < 0.05) (Fig. 4d). The only miRNA associated with osteogenesis was hsa-miR-125b-5p, and this gene was stably differentially expressed. In hPDLSCs + Pso-Exos, this gene was expressed at a 0.73-fold reduction than hPDLSC-Exos, (*P* < 0.001) (Fig. 4a, b, c). KEGG pathway enrichment analysis was performed to assess the characteristics of exosome-mediated gene expression (Fig. 4e). Related genes were more abundant in human diseases.

### Transfection effectiveness

**Fig. 5:**
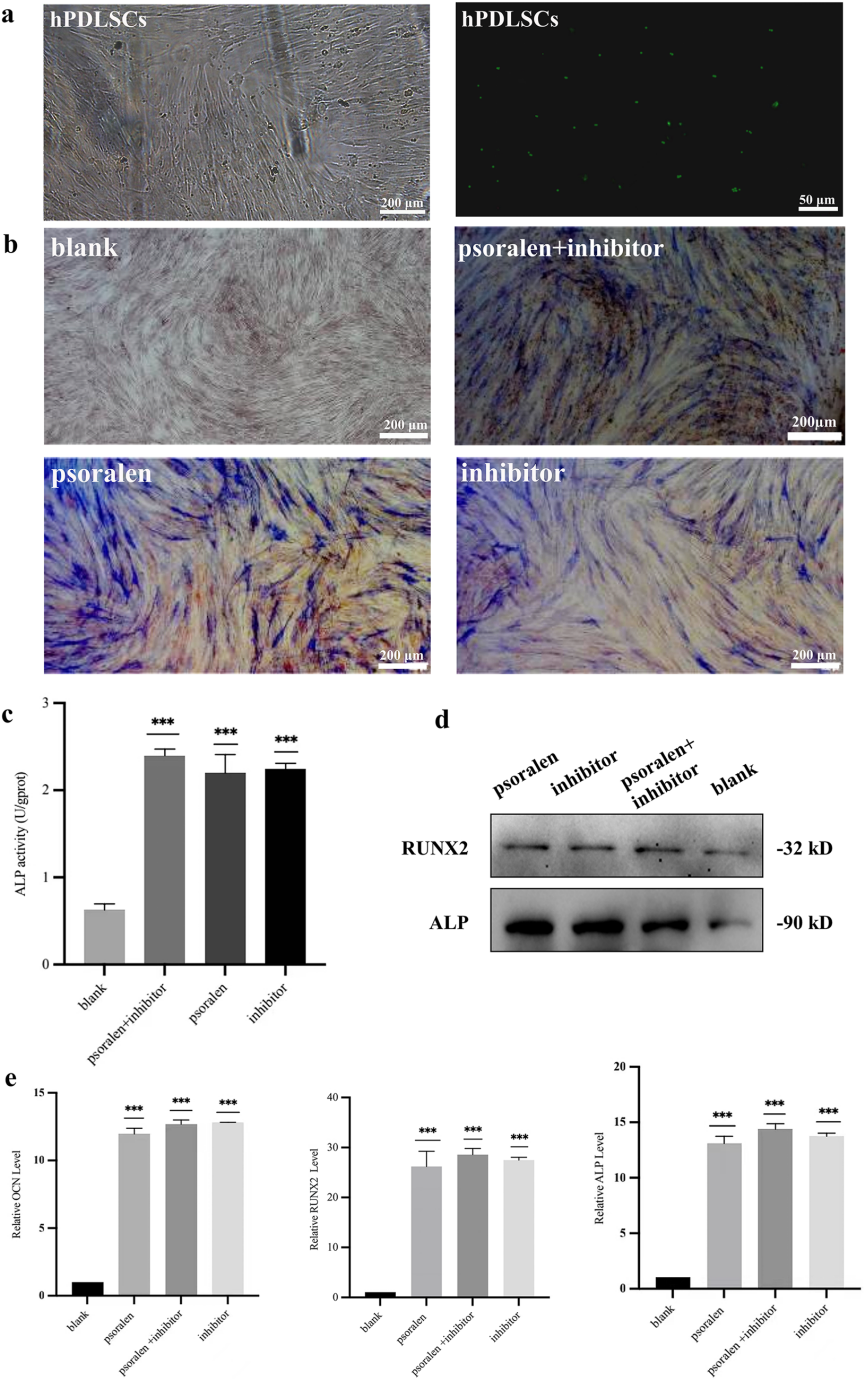
Effect of hsa-miR-125b-5p on the osteogenesis of PDLSCs **a**. Effect of inhibitor transfection. **b**. ALP staining in the blank control group;Blue‒black ALP is visible after staining of the inhibitor + psoralen group, the psoralen group, and the inhibitor group. **c**. ALP activity level. **d**. RUNX2 and ALP Western blot bands of the blank control, psoralen, inhibitor + psoralen, and inhibitor groups. e. Expression levels of OCN, RUNX2 and ALP in the blank control, psoralen, inhibitor + psoralen, and inhibitor groups

To demonstrate the level of binding of a target gene inhibitor to the target gene, the GFP gene was used to label an inhibitor, and 24 h later, observation by fluorescence microscopy showed clear green fluorescence in the nucleus, which proved that the inhibitor successfully bound to the hsa-miR-125b-5p gene to inhibit its expression (Fig. 5a).

### Effect of hsa-miR-125b-5p on PDLSC osteogenesis

The osteogenic ability of periodontal stem cells was enhanced after inhibition of hsa-miR-125b-5p expression. A blue‒black color was observed in in the inhibitor + psoralen, psoralen, and inhibitor groups after ALP staining (Fig. 5b), indicating that ALP was produced in the inhibitor + psoralen, psoralen, and inhibitor groups. The ALP activity of inhibitor + psoralen, psoralen and inhibitor groups was significantly stronger than that of blank control group (*P* < 0.001), and there was no statistical significance between inhibitor + psoralen, psoralen and inhibitor groups (Fig. 5c). Westernblot assays showed that the expression of RUNX2 and ALP (Fig. 5d) was significantly higher in the inhibitor + psoralen, psoralen, and inhibitor groups than in the blank control group (*P* < 0.001). RT‒PCR experiments showed that the expression of OCN, RUNX2, and ALP (Fig. 5e) was significantly higher in the inhibitor + psoralen, psoralen, and inhibitor groups than in the blank control group, and the differences were statistically significant (*P* < 0.001), while the differences between the inhibitor + psoralen, psoralen, and inhibitor groups were not statistically significant.

**Fig. 6:**
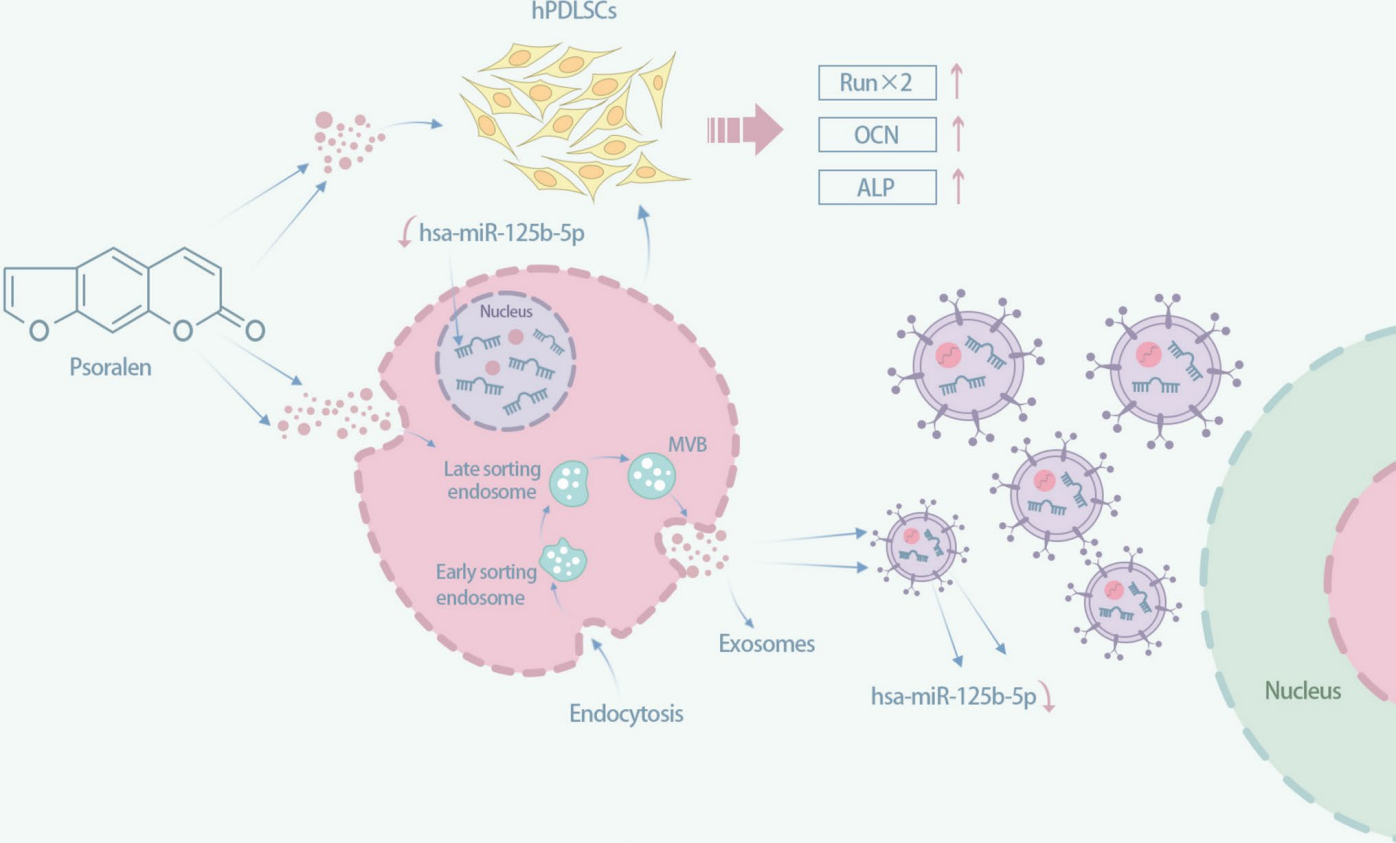
A model for the mechanism of osteogenic differentiation of periodontal stem cells regulated by psoralen

## Discussion

Periodontitis is a highly prevalent chronic oral cavity-like disease [19]. Its complex etiology is the result of a multifaceted interaction of plaque microorganisms, host immune response, and environmental and genetic factors, leading to the release of multiple enzymes and cytokines, causing progressive destruction of the gingiva, dental bone, periodontal membrane and alveolar bone, which can eventually lead to tooth loss, severely affecting normal life and placing a large financial burden on the patient [20–22].

The key to treating periodontitis is the repair and regeneration of periodontal bone tissue. Periodontal stem cells isolated from periodontal tissues show the potential to differentiate into bone, periodontal membrane and alveolar bone-like tissues and the advantage of being easier to obtain and more capable of proliferation and differentiation than other MSCs [23].

In 2004, Seo et al. first isolated PDLSCs from human periodontal tissues using enzymatic digestion, and these stem cells exhibited biological properties similar to those of MSCs. The osteogenic ability of PDLSCs was also first identified when PDLSCs were transplanted with a composite of hydroxyapatite/β-tricalcium phosphate ceramics into rats, and high expression of a large number of osteogenesis-related molecules was detected [3]. Kang et al. exploited the promotional effect of basic fibroblast growth factor (bFGF) on the proliferative capacity of PDLSCs and synergized its action with that of BMP-2 on PDLSCs to enhance the osteogenic and mineralizing capacity of PDLSCs while effectively compensating for the inhibitory effect on the osteogenesis of PDLSCs [24].Therefore, periodontal stem cells form the basis for slowing the resorption of periodontal bone tissue, promoting bone tissue repair and regeneration, and improving the prognosis of patients with periodontitis. In this study, periodontal stem cells extracted from periodontal tissue blocks were shown to carry lipogenic and osteogenic differentiation potential, and a surface antibody flow assay showed their MSC characteristics, indicating that they can be used as cell models to explore the osteogenic differentiation ability of periodontal stem cells.

Psoralen is the main component of the traditional Chinese medicine fructus psoraleae, which shows anti-osteoporotic immune, antitumor, antibacterial, anti-inflammatory and antioxidant activity. LI et al. found that psoralen stimulated osteoblast proliferation through the ERK/MAPK, JNK/MAPK and p38/MAPK pathways and that psoralen significantly increased NF-kB expression, indicating that psoralen can induce cell proliferation through the NF-kB pathway [25]. Therefore, psoralen may be a viable drug for the treatment of osteoporosis, and HUANG et al. found that psoralen promoted the differentiation of hBMSCs through the activation of the TGF-β/Smad3 pathway to accelerate osteogenesis, which may be of future value in the clinical treatment of osteoporosis [26]. These studies indicate that the osteogenic differentiation of stem cells is promoted by psoralen. In previous in vivo experiments, gavage with psoralen was found to promote alveolar bone regeneration in rats with periodontitis [9]. Some studies have confirmed that osteogenic differentiation of hPDLSCs is promoted by psoralen [10], which can be used as a potential drug for periodontal bone regeneration; however, the precise mechanism needs to be further investigated.

In the present study, 10 μg/mL psoralen was found to promote the expression of osteogenic factors such as ALP, OCN and RUNX2, confirming that psoralen induced periodontal stem cells osteogenesis, which is consistent with the results of previous studies. To further clarify the regulatory mechanism of osteogenic differentiation mediated by psoralen, the genetic information of exosomes derived from periodontal stem cells after psoralen treatment was investigated in this study.

Exosomes are extracellular vesicles secreted by different cells and found in different bodily fluids, such as blood, urine and cerebrospinal fluid, and are considered to be important transmitters of intercellular information because they are rich with a variety of biologically active proteins, DNA and miRNAs [27–30]. Exosomes from different cell sources exhibit different biological functions [31]. For example, exosomes of osteoblast origin regulate the proliferation, differentiation and maturation of osteoblasts, osteoclasts and BMSCs, thereby regulating bone resorption and bone formation [32]. CUI et al. cocultured exosomes from mineralized preosteoblasts with bone marrow stromal cells and found that osteogenic markers appeared on the surface of the bone marrow stromal cells [33]. QIN et al. found that exosomes secreted by bone marrow MSCs contained miR-196a, miR-27a and miR-206, which were involved in osteogenesis. By mediating the differentiation of osteoblasts through signaling, exosomes may promote osteoblast differentiation and stimulate bone tissue regeneration [34].Therefore, the genes carried by exosomes in each group of periodontal stem cells were subjected to high-throughput sequencing after different treatment. The results showed that 35 differential miRNAs were upregulated, and 58 differential miRNAs were downregulated, among which the level of the hsa-miR-125b-5p gene was significantly changed in the exosomes and was the only miRNA related to osteogenesis. It may be used as a target gene for osteogenic differentiation of periodontal stem cells.Studies have shown that miR22, miR210, miR299-5p and miR543 are among miRNAs that promote the osteogenic differentiation of PDLSCs [35–38], while miR125b and miR132 are among miRNAs that inhibit the osteogenic differentiation of PDLSCs [18, 39]. Mizuno et al. found that miR-125b affected osteoblast differentiation in the mouse mesenchymal layer (ST2) by regulating cell proliferation. In ST2 cells, miR-125b expression was elevated in a time-dependent manner. The elevation of miR-125b expression was attenuated in ST2 cells after BMP-4-induced osteoblast differentiation. Exogenous transfection of miR-125b inhibited ST2 cell proliferation and suppressed osteoblast differentiation. In contrast, when endogenous miR-125b antisense RNA molecules were transfected to block miR-125b, alkaline phosphatase activity was elevated after BMP-4 treatment, demonstrating that miR-125b regulated osteoblast differentiation at an early stage by inhibiting the proliferation of mouse MSCs [40]. Moreover, hsa-miR-125b-5p, a member of the miR-125b family, has been shown to be upregulated in in a high-fat environment, producing an inhibitory effect on osteogenesis.

To clarify the role of the hsa-miR-125b-5p gene, we constructed an inhibitor to verify its effect on the osteogenic differentiation of periodontal stem cells. The results showed that the osteogenic capacity of periodontal stem cells inhibited by the expression of hsa-miR-125b-5p was significantly higher than that of the blank control group, and no difference between the osteogenic ability of the inhibitor + psoralen group and the inhibitor group was found. It was hypothesized that the hsa-miR-125b-5p gene was associated with the promotion of periodontal stem cell osteogenesis by psoralen and that psoralen promoted the osteogenesis of periodontal stem cells by reducing the expression of hsa-miR-125b-5p. Differential expression of hsa-miR-125b-5p was found in the PDLSC-derived exosomes treated with psoralen.in the present study, suggesting that the hsa-miR-125b-5p gene entering exosomes with the PDLSC exosome vesicles is not highly expressed but shows the possibility of continuing to transmit osteogenesis-related information.

In conclusion, the findings revealed that the osteogenesis of periodontal stem cells was promoted by psoralen through the downregulation of hsa-miR-125b-5p expression. The downregulated hsa-miR-125b-5p was carried in exosomes derived from periodontal stem cells treated with psoralen and showed the potential to continue to transmit osteogenic information. Thus this miRNA may be a potential target gene for psoralen treatment (Fig. 6).

## Supplementary Information

Below is the link to the electronic supplementary material.

Supplementary file1 (CSV 0 KB)

Supplementary file2 (CSV 0 KB)

Supplementary file3 (CSV 0 KB)

Supplementary file4 (CSV 0 KB)

Supplementary file5 (CSV 0 KB)

Supplementary file6 (CSV 0 KB)

Supplementary file7 (CSV 0 KB)

## Data Availability

All data generated during the current study are included in the article.
